# Genomic evidence for human-mediated introgressive hybridization and selection in the developed breed

**DOI:** 10.1186/s12864-024-10259-5

**Published:** 2024-04-02

**Authors:** Heng Du, Zhen Liu, Shi-Yu Lu, Li Jiang, Lei Zhou, Jian-Feng Liu

**Affiliations:** State Key Laboratory of Animal Biotech Breeding, Key Laboratory of Animal Genetics and Breeding, Ministry of Agriculture, College of Animal Science and Technology, China Agricultural University (West District), No.2 Yuanmingyuan West Road, 100193 Beijing, China

**Keywords:** Pig, Developed breed, Genetic structure, Introgression, Selection, Modern breeding

## Abstract

**Background:**

The pig (*Sus Scrofa*) is one of the oldest domesticated livestock species that has undergone extensive improvement through modern breeding. European breeds have advantages in lean meat development and highly-productive body type, whereas Asian breeds possess extraordinary fat deposition and reproductive performance. Consequently, Eurasian breeds have been extensively used to develop modern commercial breeds for fast-growing and high prolificacy. However, limited by the sequencing technology, the genome architecture of some nascent developed breeds and the human-mediated impact on their genomes are still unknown.

**Results:**

Through whole-genome analysis of 178 individuals from an Asian locally developed pig breed, Beijing Black pig, and its two ancestors from two different continents, we found the pervasive inconsistent gene trees and species trees across the genome of Beijing Black pig, which suggests its introgressive hybrid origin. Interestingly, we discovered that this developed breed has more genetic relationships with European pigs and an unexpected introgression from Asian pigs to this breed, which indicated that human-mediated introgression could form the porcine genome architecture in a completely different type compared to native introgression. We identified 554 genomic regions occupied 63.30 Mb with signals of introgression from the Asian ancestry to Beijing Black pig, and the genes in these regions enriched in pathways associated with meat quality, fertility, and disease-resistant. Additionally, a proportion of 7.77% of genomic regions were recognized as regions that have been under selection. Moreover, combined with the results of a genome-wide association study for meat quality traits in the 1537 Beijing Black pig population, two important candidate genes related to meat quality traits were identified. *DNAJC6* is related to intramuscular fat content and fat deposition, and *RUFY4* is related to meat pH and tenderness.

**Conclusions:**

Our research provides insight for analyzing the origins of nascent developed breeds and genome-wide selection remaining in the developed breeds mediated by humans during modern breeding.

**Supplementary Information:**

The online version contains supplementary material available at 10.1186/s12864-024-10259-5.

## Background

As an important livestock species, the pig (*Sus Scrofa*) can supply staple protein to humans [1]. Domestication by ancient humans and breeding practices by modern breeders resulted in many common characteristics, such as improving productivity and adapting to hostile circumstances. However, these two processes differ in many aspects. The former domestication of pigs involved a long period of artificial selection for enhanced productivity in two discrete geographical areas, Eastern Anatolia and China, with limited introgression from other populations [2]. Whereas to achieve greater production performance, the modern breeding practice utilizes strong selection pressure on a limited number of generations of segregating populations from hybridization using two or more genetically [3, 4].

Hybridization, which may occur in many different spatial contexts, can impact both adaptation and speciation [5]. One such impact is adaptive introgression, where advantageous alleles are transmitted between different breeds [6, 7], potentially assisting the development of new hybrid breeds with superior production performance [8, 9]. Additionally, recent strong and directional positive selection has resulted in pigs better adapted towards human needs [10]. Hence, an increasing number of studies concentrate on discovering the hybridization introgression and selection in distinct pig breeds. For instance, an allele at the *AHR* gene associated with increased litter size has been transferred from Asian pigs to European commercial pigs [11], and a 14 Mb genomic region linked to adaptation to high latitude in northern Chinese pigs has been transmitted to European populations [12]. Besides, several studies also reported the genes under selection that benefit to adaptation and commercial traits of different swine breeds, like the *ELOVL3* gene with a major effect on intramuscular fat (IMF) content in some commercial pig breeds [1] and *IGF1R* gene related to the high fertility of Meishan pig [13]. However, most of the studies concentrate on long-term introgression and natural selection, the history of gene flow and artificial selection during the breeding process for many developed breeds is still unknown.

Beijing Black pig, as a typical locally developed breed cultivated in the last half century, offers an opportunity to explore the human-mediated introgression in modern breeding and the function of artificial selection during rapid breeding. Simultaneously, owing to the limitation of sequencing technology in the past, the genome architecture and the population origin of Beijing Black pig have not been fully discovered. Beijing black pig is renowned for its exceptional combination of traits derived from Chinese indigenous pig breeds, such as superior meat quality, robust disease resistance, and desirable reproduction performance, as well as characteristics from European commercial pig breeds including fast growth rate, high lean meat content and efficient feed conversion [14]. This breed offers a valuable model for investigating its genomic composition and identifying the candidate genes that underlie its advantageous traits, which can provide new insights into understanding the genomic architecture of nascent developed breeds and assist in the development of new hybrid breeds in the future.

To explore the introgressive hybridization and the artificial selection during the breeding process, Beijing Black pig and two of its ancestors (Yorkshire and Shenxian pig) were sampled and sequenced in this study. After conducting population genetic structure analysis, introgression analysis, and selection signature analysis, we not only detected the genomic evidence of Beijing Black pig’s introgressive hybridization formation but also disclosed a phenomenon that human-mediated introgression can shape the porcine genome structure. Besides, we recognized the genomic regions under selection in Beijing Black pig and identified two important genes related to meat quality traits. Our research provided a model for analyzing the origins of nascent developed breeds as well as genome-wide selection remaining in developed breeds mediated by humans during modern breeding.

## Results

### Population genetic structure of Beijing Black pig

Beijing black pig is a prominent locally developed black pig breed in China with a wide range of lineages. Its breeding history showed that it originated from the crossbred between Asian indigenous pigs like Shenxian pig and Dingxian pig (extinct) and European commercial pig breeds including Yorkshire. To validate the consistency between the population genetic structure of the Beijing Black Pig and its breeding history, we conducted whole genome sequencing of 100 Beijing Black pigs. Additionally, nine Eurasian representative breeds were sequenced or collected from the public dataset in this study, including 38 Shenxian pigs, 40 Yorkshire pigs, five Iberian pigs, five Mangalica pigs, five Duroc pigs, five Landrace pigs, five Bama Xiang pigs, five Wuzhishan pigs, and five Hetao pigs. The sequencing coverage depth for the above 213 pigs ranged from 10.59 × to 57.44 ×, and the average depth is 20.65 × (See Additional file 1, Table S1). Using the Sscrofa11.1 reference genome, a total number of 48,544,863 high-quality single nucleotide polymorphisms (SNPs) were identified in these pig breeds.

**Fig. 1:**
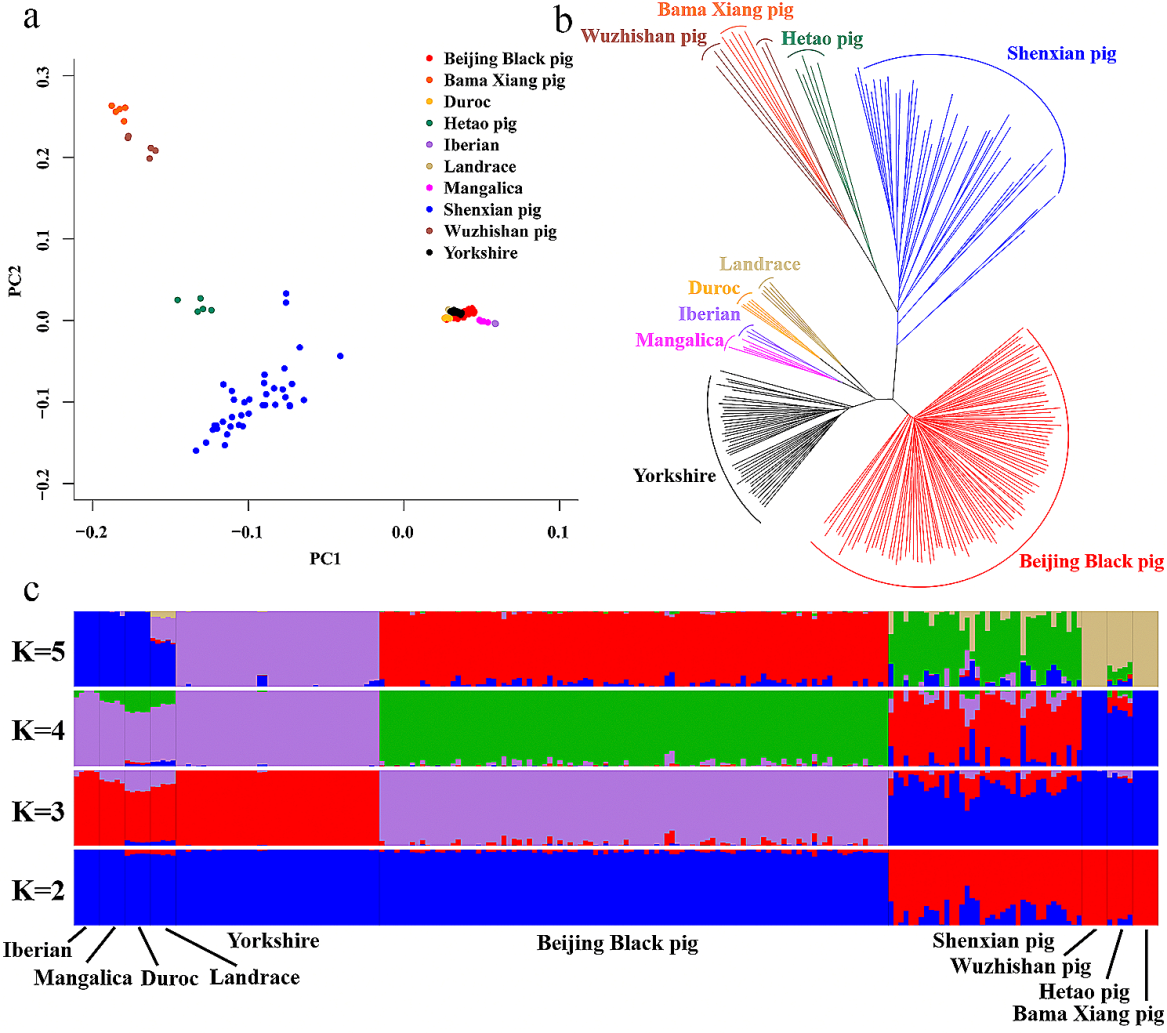
Population genetic structure of Beijing Black pig. (**a**) Principle component analysis for the first two PCs of 10 pig breeds. (**b**) Neighbor-joining phylogenetic tree constructed from SNV data among 10 populations. (**c**) Structure analysis with K assumed ancestral groups (K = 2–5)

To infer the phylogenetic relationships of the above breeds, we performed principle component analysis (PCA) based on SNPs identified in these breeds across the whole genome. The PCA results could distinguish European and Asian populations (Fig. 1a). Furthermore, the phylogenetic tree was constructed utilizing the neighbor-joining (NJ) method based on the pairwise genetic distances of whole-genome SNPs (Fig. 1b). We further conducted a population structure analysis, estimating individual ancestry along with admixture proportions under the assumption of K ancestral populations (Fig. 1c). K = 5 represented the optimal number of assumed ancestors by cross-validation error test (See Additional file 3, Figure S1). Beijing Black pig shared genetic information with European pig and partial Asian pig when K was set to 2, which indicated the genome of Beijing Black pig may contain the regions influenced by Eurasian pigs. Beijing Black pig was separated from the other breeds when K = 3, 4 or 5, which indicated that Beijing black pig has formed a unique genetic structure after multiple generations of breeding and can be used as an independent genetic resource. The PCA, phylogenetic tree, and population structure analyses suggested that the genetic divergence between Asian pigs and Beijing Black pigs was greater than that between European pigs and Beijing Black pigs.

We further separately calculated the *Fst* values between Beijing Black pig and the other breeds based on 48,544,863 SNPs. The result (See Additional file 1, Table S2) showed that among Asian breeds, the Shenxian pig is the closest to the Beijing Black pig, while among European breeds, the Yorkshire is the closest to the Beijing Black pig, which corresponded to the documented breeding history that Shenxian pig and Yorkshire are the ancestor populations during the cross-breeding process of Beijing Black Pig.

### Shared variants between developed breed and its ancestral breeds

Compared to its ancestors (Yorkshire and Shenxian pig), Beijing black pig has reflected significant differences in production performance (See Additional file 1, Table S3). To fully explore the genetic composition of Beijing black pig and accurately detect genomic footprints left by the selection, we further detected whole genome SNPs in these three pig breeds. A total number of 33,028,690 high-quality SNPs were identified (See Additional file 3, Figure S2a), including 91,907 non-synonymous mutations (See Additional file 3, Figure S2b). We observed 20.50 million SNPs shared between Beijing Black pig and Shenxian pig (the SNPs detected in both two breeds), which exceeded that shared between Beijing Black pig and Yorkshire (16.16 million) or between Yorkshire and Shenxian pig (16.46 million). Interestingly, even though the number of shared SNPs between Beijing Black pig and Shenxian pig was significantly more than the other pairs, the pairwise *Fst* value between Beijing Black pig and Yorkshire was the lowest (0.1568) among the three comparison pairs (See Additional file 3, Figure S2c). We further separately calculated the *Fst* values between Beijing Black pig and the other two breeds using three SNP datasets, including SNPs detected in the three breeds and SNPs only detected in Beijing Black pig and Shenxian pig or Yorkshire. The results analyzed on the three SNP datasets indicated the same trend that the *Fst* value between Beijing Black pig and Yorkshire was smaller than the value between Beijing Black pig and Shenxian pig (See Additional file 1, Table S4). From the perspective of *Fst* values, the genetic differentiation between Beijing Black pig and Yorkshire was less than that between Beijing Black pig and Shenxian pig.

### Phylogenetic analysis and pervasive inconsistent gene trees

**Fig. 2:**
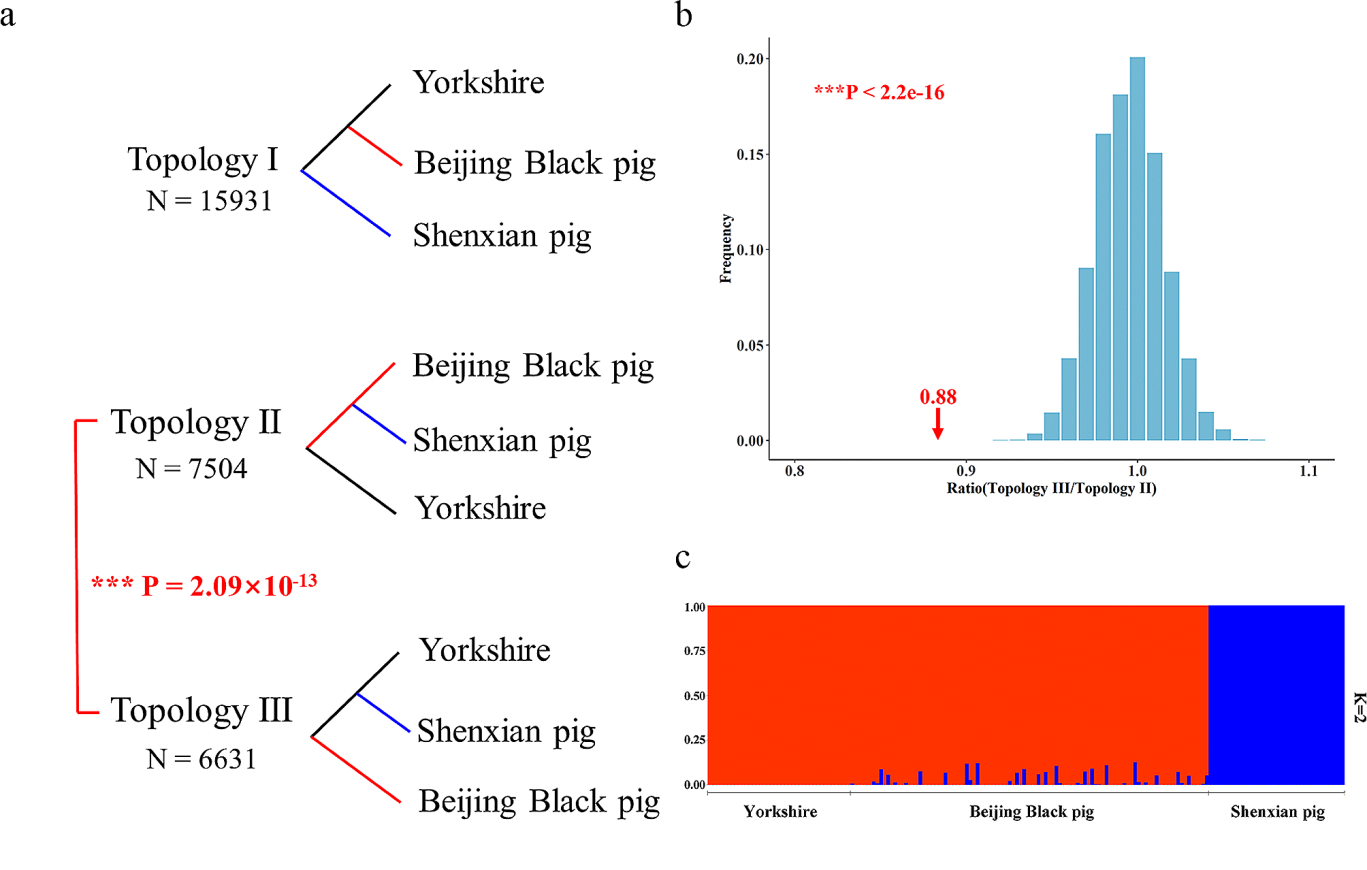
Pervasive inconsistent in gene-tree phylogenies. (**a**) Phylogenetic topologies and the corresponding numbers, the number for Topology II was significantly more (*P* = 2.09e-13) than that for Topology III. (**b**) Simulations under solely ILS scenario. The red arrow indicates the observed ratio (Topology III/Topology II) from 30,066 ortholog groups. The blue bars are a histogram of the ratios obtained under the ILS scenario. The solely ILS hypothesis was strongly rejected due to a significant difference (*P* < 2.2e-16) between observed and simulated ratios. (**c**) Individual ancestry for Beijing Black pig population using the supervised method

To further validate our inferences of the phylogeny for Beijing Black pig, Yorkshire, and Shenxian pig, we downloaded sequencing data of 6 warthogs (See Additional file 1, Table S1) as the outgroup and generated a coalescent-based species tree based on 30,066 genes. The coalescent-based species tree agreed with the previous NJ tree. However, three distinct gene topologies were obtained based on SNPs from the 30,066 genes. The most common tree (from 15,931 genes) was in accordance with the species tree and indicated Shenxian pig as a sister clade to the clade consisting of Beijing Black pig and Yorkshire (Topology I). However, the other topologies covered almost half (47.01%) of genes and revealed that Beijing Black pig was a sister clade to Shenxian pig (Topology II) significantly more (χ^2^ test, *P* = 2.09e-13) than the number for topology III, in which Beijing Black pig clustered as a separate lineage (Fig. 2a). The unequal proportions of the three topologies further demonstrated the hybrid origin of Beijing Black pig, since the latter two (Topologies II and III) would be expected to be nearly equal under a solely incomplete lineage sorting (ILS) scenario [15, 16]. To further examine these significant differences, we simulated the gene trees and calculated the proportion of each topology under the effects of ILS. The solely ILS hypothesis was strongly rejected due to a significant difference (*t*-test, *P* < 2.2e-16) between observed and simulated ratios (Topology III/Topology II) (Fig. 2b). Furthermore, we estimated the individual ancestry for all individuals in Beijing Black pig population using a supervised method to recognize whether Beijing Black pig was introgressed lineage or homoploid hybrid speciation (HHS) lineage. The results indicated that only a few individuals exhibited genetic admixture (Fig. 2c), which suggested the introgression hybridization referred to the form of Beijing Black pig rather than HHS. Hence, Beijing Black pig can be used to further analyze the introgression genomic region obtained from its ancestry with potential function.

### Shenxian pig introgression into Beijing Black pig

**Fig. 3:**
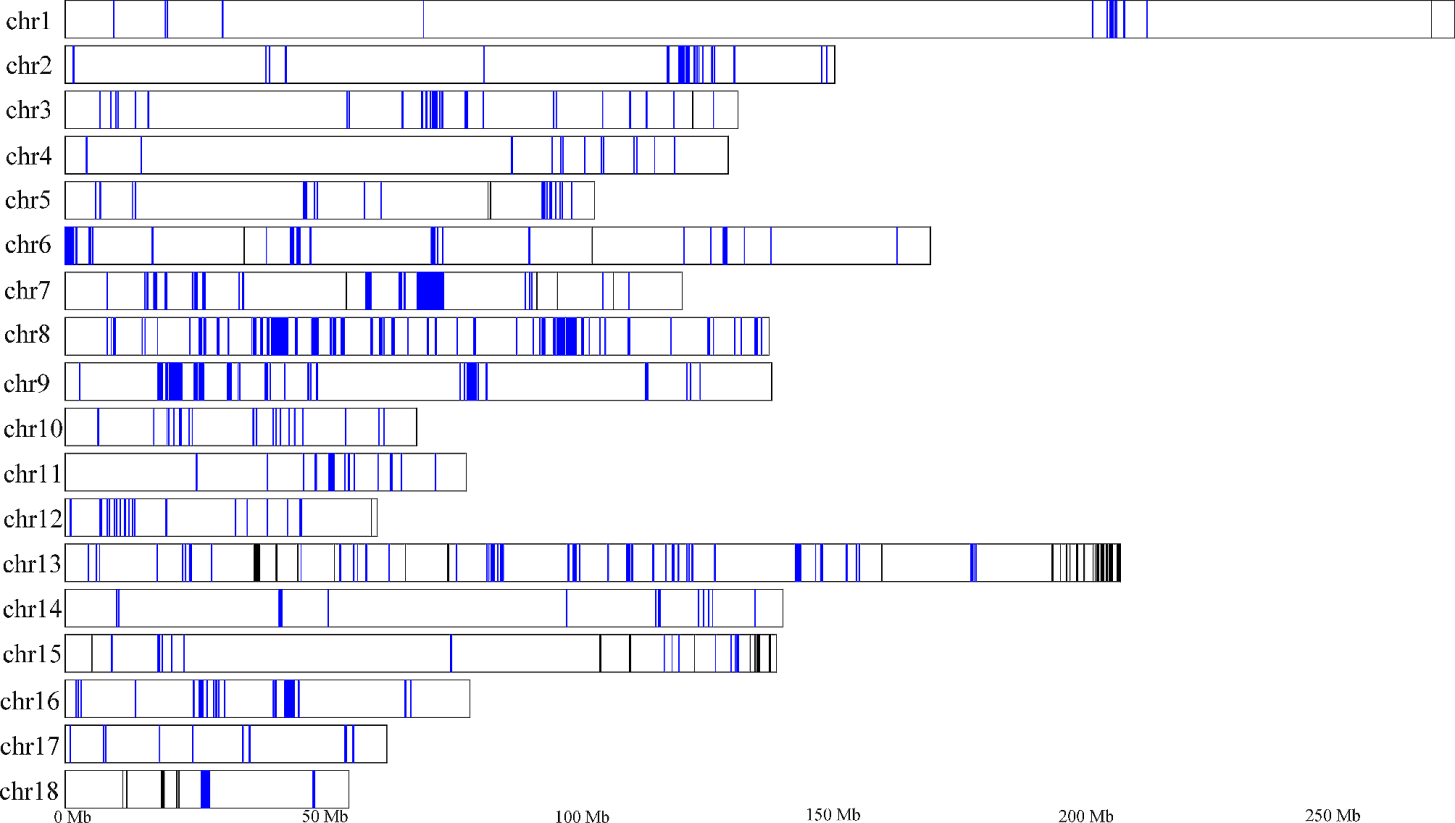
Shenxian-pig genome introgression into Beijing-Black-pig and high similarity genomic regions of Beijing-Black-pig to Yorkshire. Blue lines display introgression from Shenxian pig to Beijing black pig across the genome. Black lines display high similarity genomic regions of Beijing Black pig to Yorkshire

Although the population structure and phylogenetic analysis suggested that the genetic difference between Beijing Black pig and Yorkshire was smaller than Shenxian pig, the gene trees analysis also indicated that some genomic regions in Beijing Black pig exhibited closer genetic relationships with Shenxian pig. Hence, we carried out investigations to clarify the nucleotide distances (*d*_*xy*_) between populations of different genomic regions and discovered 13.57% of Beijing Black pig genomic regions had lower *d*_*xy*_ with Sehnxian pig compared to Yorkshire (See Additional file 3, Figure S3), which indicated potential introgression in genomics between Beijing Black pig and Shenxian pig. To test this hypothesis, we used the ABBA-BABA test and calculated the *D* statistics. *D* statistics test was applied following the tree topology ((Yorkshire, Beijing Black pig), Shenxian pig, Warthog), and the results suggested that Beijing Black pig was possibly introgressed from Shenxian pig, producing a significant *Z* score of -4.625. Then, we performed a population-based introgression analysis to estimate the magnitude of gene flow from Shenxian pig into Beijing Black pig. The *f*_*d*_ statistic, which suggested gene flow when 0 < *f*_*d*_<1, was used to calculate the fraction of introgression in Beijing Black pig in 50 kb windows (See Additional file 3, Figure S4). Simultaneously, we also inferred the local ancestry inference across the genome and selected the top 5% windows with the high ancestry of Shenxian pig (See Additional file 3, Figure S5). The overlap of introgression regions detected by *f*_*d*_ and regions with high ancestry of Shenxian pig were considered as Shenxian pig derived introgression into Beijing Black pig (Fig. 3).

We observed that 554 regions occupied 63.30 Mb that were most likely of Shenxian pig origin, and 613 functional genes overlapped with these regions. Moreover, in these genes, the amount of Topology I gene trees was no more than Topology II (Topology I/ Topology II ratio was 0.96), which significantly contrasted with the pattern of the whole genome (Topology I/ Topology II ratio was 2.12). These genes enriched in 23 Kyoto Encyclopedia of Genes and Genomes (KEGG) pathways (See Additional file 1, Table S5), most were associated with commercial traits like meat quality (MAPK signaling pathway and Rap1 signaling pathway), fertility (oocyte meiosis pathway) and disease-resistant (herpes simplex virus 1 infection).

Chromosomes 6 and 9 contain the longest two consecutive regions of inferred introgression in the genome of Beijing Black pig (See Additional file 1, Table S6). The longest region in Chromosome 6 comprised 54 genes enriched in three Gene Ontology (Go) terms. Especially, we noticed that the *ACSF3* gene, which is associated with lipid metabolism traits [17, 18], was in the longest introgression region, and the Topology II gene tree of this gene also revealed that this gene may share more haplotypes with Shenxian pig. Besides, in the 1.45 Mb longest consecutive region of chromosome 9, only two functional genes were identified, one of which was the *NDUFA4* gene. This gene plays an important role in oxidative phosphorylation [19] and was reported as a candidate gene related to the IMF trait [20, 21]. It can be inferred that advantageous genes related to meat quality and carcass traits of Shenxian pig were retained during the breeding process of Beijing black pig, which explains why Beijing black pig is more similar to Shenxian pigs in terms of meat quality and carcass traits.

### High similarity genomic regions of Beijing Black pig to Yorkshire

**Fig. 4:**
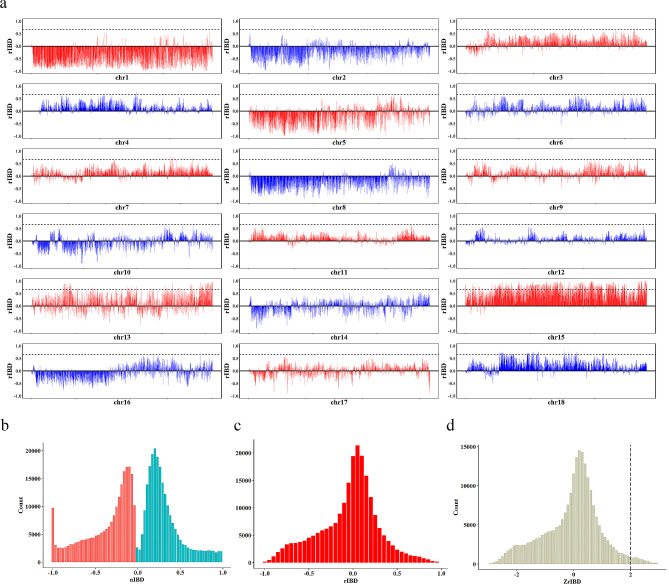
Distribution of regions in the genome where Beijing Black pig contains introgressed haplotypes from Yorkshire. (**a**) X-axis represents 18 autosomes, and the y-axis represents the relative frequency of Beijng Black pig haplotypes IBD with Shenxian pig or Yorkshire, ranging from − 1 to 1. The dotted line represents the threshold for extreme IBD with Yorkshire compared with Shenxian pig. (**b**) Distribution of the relative proportion of IBD haplotypes in Beijing Black pig and Shenxian pig (red, -1 to 0) or Yorkshire (blue, 0 to 1) in bins of 10,000 bp. (**c**) Distribution of the rIBD scores for Beijing Black pig haplotypes. (**d**) Z-transformed distribution of rIBD. The red line represents the threshold for extreme IBD with Yorkshire compared with Shenxian pig

Since Yorkshire is the ancestral origin of Beijing Black pig, it can be inferred that they may share the same haplotypes. Because of the limitation of the species tree topology, we employed relative identical by descent (rIBD) to estimate the fraction of Yorkshire haplotypes in Beijing Black pig. In brief, we first identified local regional haplotypes that were IBD to individuals between Beijing Black pig and the other two populations. Then, the numbers of observed IBD tracts between populations were normalized from 0 (no IBD identified) to 1 (IBD shared by all individuals within the populations). The normalized IBD between Beijing Black pig and Yorkshire (nIBD(Yorkshire)), and between Beijing Black pig and Shenxian pig (nIBD(Shenxian pig)), were used to calculate the rIBD. Finally, a threshold of two standard deviations from the mean in the Z-transformed rIBD distribution assisted us to identify the genomic regions in Beijing Black pig more likely to be Yorkshire (Fig. 4a and d). Simultaneously, we excluded the regions that were outside the top 5% windows with the highest ancestry of Yorkshire in the previous local ancestry inference analysis. In summary, we observed 372 10-kb bins with an average nIBD value > 0.80 and eventually merged 106 regions (See Additional file 1, Table S7) with lengths ranging from 10 kb to 270 kb, which were high likely to be Yorkshire (Fig. 3). These regions contained 68 genes that were enriched in 22 KEGG and Reactome pathways (See Additional file 1, Table S8). Especially for the two largest consecutive regions with high similarities to Yorkshire on Chromosome 13, we identified a crucial functional gene, the *DSCAM* gene, which was enriched in the developmental biology pathway. Previous studies reported that the *DSCAM* gene played an important role in balancing developmental mechanisms and might be an important candidate gene for residual feed intake [22, 23]. It can be inferred that the breeding process of Beijing black pig selectively retained favorable genes associated with the feed conversion efficiency of Yorkshire, which could explain why Beijing black pig resembles Yorkshire pig more in terms of growth traits.

### Candidate genes for meat quality traits under positive selection in Beijing Black pig

**Fig. 5:**
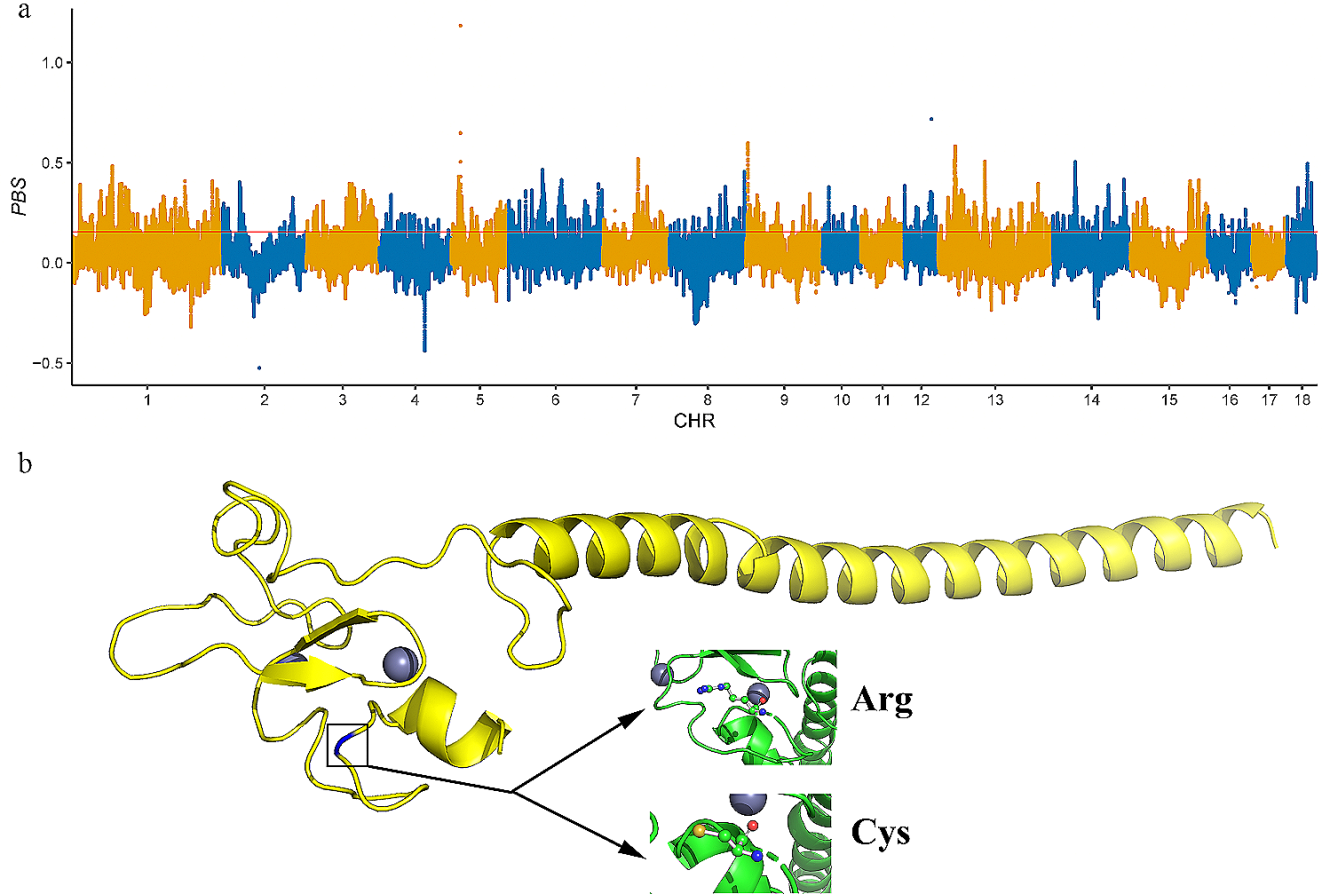
Selection signatures and protein structure prediction of Beijing black pig. (**a**) Genome-wide distribution of selection signatures detected by PBS. X-axis represents 18 autosomes, and Y-axis represents PBS statistic values. Red line displays the threshold level of 5%. (**b**) Prediction of protein conformation space for RUFY4 generated by missense SNV (rs322504869)

PBS analysis was performed to detect the genomic selective sweep signals in Beijing black pig by comparing them with Yorkshire and using Shenxian pig as the outgroup. The PBS value was calculated in 50 kb windows with 2 kb steps along the whole genome. PBS values ranged from − 0.5251 to 1.1841, with a mean of 0.0450. After filtering windows coincided with genomic introgression, the top 5% of PBS windows (threshold: 0.154713, Fig. 5a) were selected. As a result, a total of 53,672 remaining windows, which account for 7.77% of whole genomic regions, were considered potential candidate regions under positive selection.

**Fig. 6:**
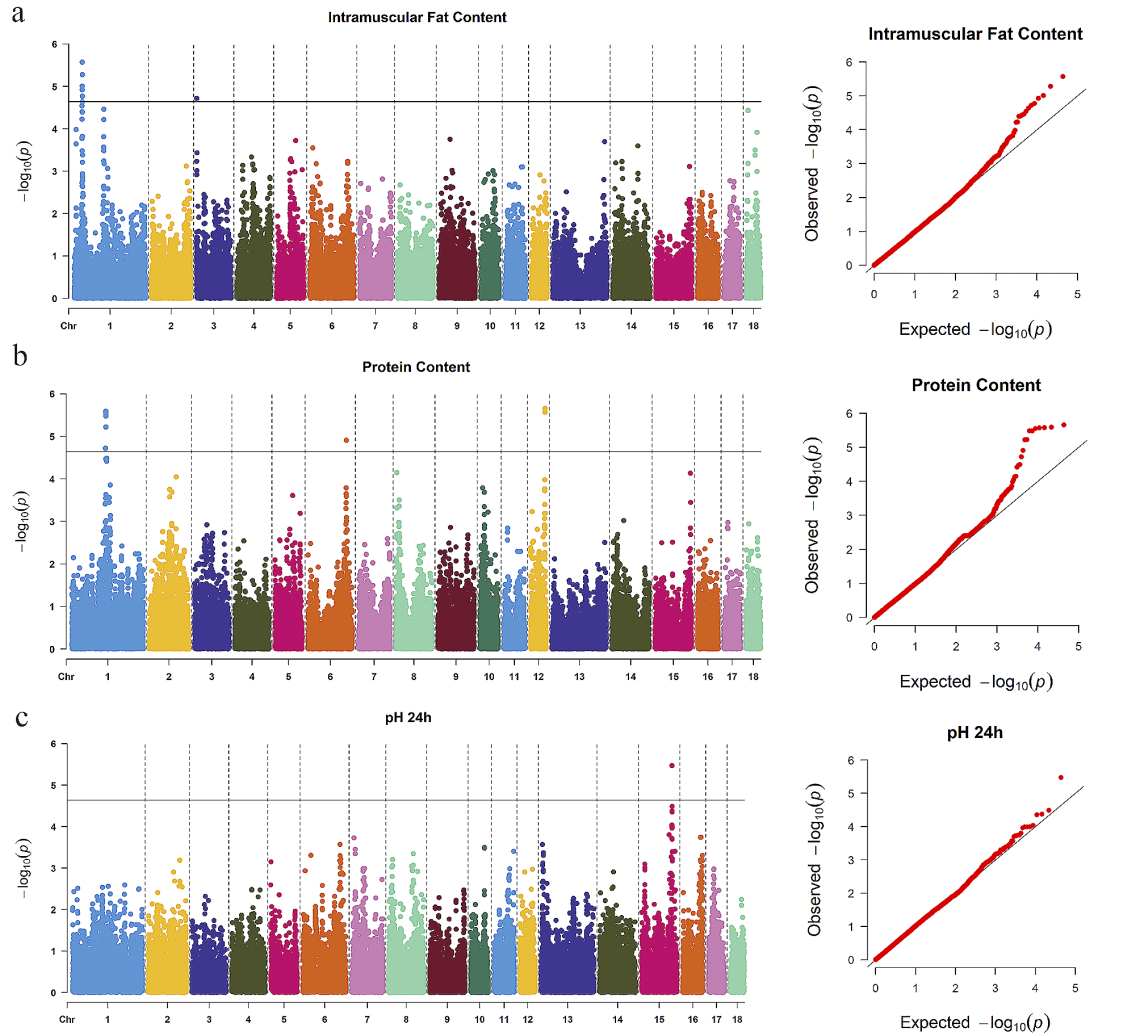
Manhattan plots and Q-Q plots for meat traits GWAS of Beijing Black pig. (**a**): IMF content; (**b**): protein content; (**c**): pH 24 h

Since significant SNPs identified by genome-wide association study (GWAS) are more likely to occur in the vicinity of the regions under selection, we further performed GWAS on the meat quality trait of Beijing Black pig. Meat quality traits include intramuscular fat (IMF) content, protein content, and pH 24 h (See Additional file 1, Table S9). Finally, a total of 21 significant SNPs (See Additional file 1, Table S10) and four linkage disequilibrium (LD) blocks (See Additional file 3, Figure S6) associated with meat quality traits were identified by GWAS (Fig. 6).

To further identify the candidate genes related to meat quality traits under selection in Beijing Black pig, we integrated the results of PBS as well as GWAS and discovered two overlapped core regions (Chromosome 6: 146,999,505–147,018,965 and Chromosome 15: 119,984,036–120,286,163). Two genes, *DNAJC6* and *RUFY4* gene, related to meat quality, were detected in the core regions. Previous research indicated that *DNAJC6* was related to IMF content and fat deposition [24–26], whereas *RUFY4* was considered as a candidate gene affecting meat pH and tenderness [27–29]. Moreover, in Beijing Black pig populations, 16 missense SNVs (See Additional file 1, Table S11) in *DNAJC6* and *RUFY4* were uncovered. *RUFY4* (RUN and FYVE domain containing 4) gene, a member of the RUFY family, was reported to interact with PtdIns(3)P in membranes and interact with Rab7 to promote autophagy [30]. Particularly, we found a missense SNV (exon12:c.C1669T: p.R557C; rs322504869 in the Ensembl database) within the *RUFY4* that generated a gain of Zinc binding, leading to a change in the protein conformation space (Fig. 5b). Therefore, identifying this nonsynonymous mutation in *RUFY4* helps us better explain the meat quality of Beijing Black pig.

## Discussion

In this study, we found out an interesting phenomenon that Beijing Black pig as an Asian locally developed breed has a tightly genetic relationship with European commercial pigs than Asian indigenous pigs. Although the SNPs detected in both Beijing Black pig and Yorkshire were less than in Beijing Black pig and Shenxian pig, contrarily, the genetic relationship between Beijing Black pig and Yorkshire showed closer than the Shenxian pig. We divided the SNPs both in Beijing Black pig and the other two breeds into three parts, including the SNPs detected in the three breeds and the SNPs only detected in two breeds. The *F*_*st*_ results for these three SNP datasets were in accordance with all SNPs in the three breeds. This might be because the differences between the allele frequencies of shared SNPs in Beijing Black pig and Shenxian pig were larger than the differences between Beijing Black pig and Yorkshire. We speculated that Beijing Black pig had greatly influenced by Shenxian pig in the initial developed period. However, in the following human-mediated breeding, the allele frequencies of these SNPs were selected that tend to be far away from the frequencies of Shenxian pig. Unlike the indigenous pigs, whose genetic relationships correspond to their spatial distribution, human-mediated breeding contributed to this special genomic architecture of Beijing Black pig. This is different from many usual introgressive hybridization models that transmit beneficial alleles from introduced breeds to local breeds by adaptive introgression, and most genomic regions of new breeds are still similar to local breeds. However, in this model, most genomic areas of this hybrid breed have a high similarity to the introduced breed, and only a small part of alleles that came from the local breed has remained. This indicates that human-mediated breeding may significantly influence the genome structure of nascent developed breeds in a divergent approach compared to hybridization without human intervention and purposefully keep useful alleles derived from ancestors. Hence, it is important to study the hybrid breeds with stable heritability and disclose their genomic architecture to further understand the function of modern breeding in shaping their phenotypes.

Evolutionary events such as introgressive hybridization, HHS, and ILS often complicate our inferences of phenotypic evolution by causing phylogenetic incongruence between morphological and molecular data [31–33]. For example, the bodily form of Beijing Black pig is more likely to Yorkshire, while Beijing Black pig shares various meat quality characteristics with Shenxian pig. It is challenging to discern the genetic relationship between the Beijing Black pig and its two ancestral populations based solely on phenotype. Both the phylogenetic tree based on whole genome SNPs and the coalescent-based species tree based on 30,066 genes indicated Beijing Black pig and Yorkshire clustered as a clade and sister to Shenxian pig. Moreover, our analysis of multiple gene sets revealed highly discordant gene topologies for the three populations, which might have resulted from introgression, HHS, or ILS. Further, after dissecting the ratio of these distinct gene topologies, we excluded ILS as an explanation due to the significant difference between Topology II and III. Previous research reported that for three taxa from a common ancestral species with random mating, shared variations concordant with the species tree should be the most abundant, while the other two discordant with the species tree should share equally in bifurcating speciation due to ILS [15, 16]. Furthermore, the ABBA-BABA test, which has been proven to be a powerful method for detecting hybridization also rejected the ILS hypothesis. Moreover, the supervised admixture analysis for Beijing Black pig revealed that only a few individuals exhibit genetic admixture. Previous studies concluded that introgression may produce genetic admixture in a few individuals or populations, rather than in all hybrid offspring, as HHS does [34–36]. Our results validated the occurrence of introgression hybridization during the breeding process of Beijing Black pig using genomic data and implied that genomic data can track the recent hybridization incidents in developed breeds.

Hybridization and introgression offer the opportunity for the exchange of genetic material. In this study, we identified the introgressed haplotypes in Beijing Black pig and their potential function after validating Shenxian pig-derived introgression in Beijing Black pig. The candidate introgression locus contained 613 functional genes, which were enriched in commercial traits related pathways such as the MAPK signaling pathway and the Rap1 signaling pathway corresponding to meat quality [37–40], the oocyte meiosis pathway regulating fertility [41, 42], and the herpes simplex virus 1 infection pathway affecting the ability of disease-resistant [43, 44]. Moreover, we discovered two longest introgression regions. Although the length difference between the two regions is only 50 kb, the number of genes contained in the two regions differs by a factor of four. The genes in 1.50 Mb introgression regions of Chromosome 6 can be used to study the gene function for the similarity phenotypes between Beijing Black pig and Shenxian pig. Even though only two functional genes were discovered in the longest introgressed haplotype of chromosome 9, one important gene, the *NDUFA4* gene, was identified. Previous studies report that this gene plays an important role in oxidative phosphorylation [19] and is expected as a candidate gene related to IMF content trait [20, 21]. This gene will require further functional tests and be considered as a candidate gene applied in the future breeding of meat quality traits for newly developed breeds.

It is well known that artificial selection has greatly shaped pig genomes during the process of pig breeding [45]. With the development of sequencing, various methods have been developed for detecting different types of selection signatures. These methods contained using haplotype structure, summary statistics of allele frequency distributions, population differentiation and expectations from mathematical models [46]. All these methods detect different types of candidate genomic regions under selection. In this study, to dissect the potential genomic regions of Beijing Black pig under selection after diverging from Yorkshire, we applied the PBS method to detect the candidate regions. The PBS method was proposed to detect a significant change in allele or haplotype frequency along the lineage of one population after it diverged from other populations. Many previous studies demonstrated its efficiency in detecting candidate selection signatures in a target population over short divergence times [47–49]. We finally identified 53,672 candidate selection windows specific to Beijing Black pig and discovered that *DNAJC6* and *RUFY4* are related to meat quality traits.

More importantly, there is a consensus that the ultimate selection plays a central role in the integration of introduced alleles into the recipient genome. In this study, we performed PBS and GWAS analysis to identify candidate genes for meat quality traits under positive selection in Beijing Bl ack pig. Using the PBS method to compare the pairwise *F*_*st*_ values between three populations, we can estimate the frequency change that occurred in Beijing Black pig since its divergence from Yorkshire. Considering that meat quality traits are the most prominent phenotypic characteristic of Beijing black pig, GWAS on meat quality traits was performed to reflect the important imprints of artificial selection in Beijing black pig. Genes overlapped by PBS and GWAS analysis represent strong candidates for the genetic basis of meat quality. *DNAJC6* and *RUFY4* were identified in this study as important genes associated with meat quality traits. Previous research indicated that *DNAJC6* is related to IMF content and fat deposition [24–26], and *RUFY4* was considered as a candidate gene affecting meat pH and tenderness [27–29]. Besides, an important missense SNV (rs322504869) within the *RUFY4* was detected, generating a gain of Zinc binding, leading to a change in the protein conformation space. Although missense SNVs were primarily targeted, mutations in intronic and UTR were also included in this study. Selection may have acted directly on these variants or other linked non-coding variants to influence the regulation of candidate genes. Further molecular studies will be needed to investigate the direction and magnitude of gene expression changes associated with these SNVs, the tissues and developmental time points affected, and the downstream target genes that show altered regulation.

## Conclusions

In this study, we provided comprehensive large-scale sequencing for a locally developed breed, Beijing Black pig, and investigated ancestry tracts in its genome from origin populations. Our results demonstrated that the hybridization between European and Asian domestic pigs occurred during the formation of Beijing Black pig. We also identified candidate ancestry regions in Beijing Black pig, including MAPK signaling pathway and Rap1 signaling pathway related genes in haplotypes of Shenxian pig origins and developmental biology pathway related genes in haplotypes of Yorkshire origins. Finally, we discovered two regions under selection linked to meat quality. On the whole, this study supplied a valuable model for exploring ancestry origin tracts in developed breeds and their effects on traits, as well as the genomic footprints under selection after divergence from the last diverging lineage.

## Methods

### Data collection

Ear samples were sampled from 78 Beijing Black pigs and 38 Shenxian pigs. Genomic DNA was isolated from these samples with the use of the TIANamp Genomic DNA Kit (TIANGEN, Beijing, China). Libraries construction for the sequencing of Beijing Black pig were according to the protocol of MGISEQ, and sequencing was performed on MGISEQ 2000 platform (MGI, Shenzhen, China) with 150-bp paired-end reads. Meanwhile, libraries of Shenxian pigs were prepared depending on the DNBSEQ library prepping protocols, and sequencing was used DNBSEQ-T7 (DNB, Shenzhen, China) with 150-bp insert size. Simultaneously, sequencing data of 68 individuals (including 40 Yorkshire pigs) analyzed in this study were achieved from the public database (See Additional file 1, Table S1).

### Genomic read mapping and variants calling

For each sample, the paired-end reads were filtered by TrimGalore (v0.6.1) [50] to remove adapter sequences and low-quality reads. BWA-MEM (v0.7.17) [51] was employed to align the filtered reads to the Sscrofa11.1 reference genome. SAMtools (v1.15) [52] was used to sort the mapped reads, and samblaster (v.0.1.26) [53] was applied to mark potential PCR duplications. Then, to obtain the hard-called variants in explored populations, we adopted the GATK [54] (4.1.2.0) HaplotypeCaller best practice. SNPs were filtered using the VariationFiltration in GATK, according to the following criteria: (1) approximate read depth > 10×; (2) variant confidence/quality by depth > 2.0; (3) RMS mapping quality (MQ) > 40.0; (4) Phred-scaled P value using Fisher’s exact test to detect strand bias < 60.0; (5) Z-score from the Wilcoxon rank sum test of Alt vs. Ref read MQs (MQRankSum) > − 12.5; and (6) Z-score from the Wilcoxon rank sum test of Alt vs. Ref read position bias(ReadPosRankSum) > − 8.0. Then, the high-quality SNPs were processed for gene-based annotations using the ANNOVAR (v2020-06-08) [55] software, for which the corresponding gene annotation file was downloaded from the Ensembl 107.

### Population genetic analysis

The NJ tree was constructed for the whole-genome SNPs by MEGA (v11) [56] according to the pairwise genetic distances calculated by emmax (beta-07Mar2010) [57]. Principle component analysis (PCA) was conducted using the GCTA (v1.93.2) [58]. ADMIXTURE (v1.3.0) [59] was used to perform the unsupervised and supervised clustering analysis. We increased the number of predefined genetic clusters from K = 2 to K = 10 for the unsupervised genetic structure analysis. When the supervised clustering analysis was executed, the K was set at 2.

### Test for hybridization based on the whole genome

We performed the hybridization tests between the three breeds, Beijing Black pig, Yorkshire, and Shenxian pig, with Warthog as the outgroup. The porcine genes annotated in Ensembl 107 were employed in this analysis. Then, SNPs of each gene were extracted to construct gene trees. The genes with no more than ten SNPs were removed. IQ-TREE (v1.6.12) [60] was employed to construct an ML tree for each gene. Using the 30,066 produced gene trees, ASTRAL (v5.7.1) [61] was used to estimate the species tree under a multi-species coalescent model. Combined with the previous species tree, we used DendroPy (v4.5.2) [62] to simulate the gene trees under the ILS scenario.

### Population admixture analysis and introgression analysis using ABBA-BABA tests

We calculated *d*_*xy*_ as follows:

$$ {d}_{xy}=\frac{1}{{n}_{x}{n}_{y}}\sum _{i=1}^{{n}_{x}}\sum _{j=1}^{{n}_{y}}{k}_{ij}$$

where *n*_*x*_ and *n*_*y*_ correspond to the number of individuals in populations *x* and *y*, and *k*_*ij*_ corresponds to the number of differences between the *i*th (from population *x*) and *j*th (from population *y*) haplotypes.

We used *D* statistics to test and quantify admixture in the studied three populations. *D* statistic was computed by qpDstat function of ADMIXTOOLS (v7.0.1) [63], and its value was used to evaluate gene flow between different porcine populations. Under a given four-taxon topology *D* ((P1, P2), P3, O), a significant positive value indicated gene flow between P1 and P3, while a significant negative statistic indicated gene flow between P2 and P3. However, the *D* statistics only reflected the whole genome introgression; we also calculated the *f*_*d*_ statistics to estimate the proportion of introgression in a given window. In contrast to the *D* statistics, under the given four-taxon topology *f*_*d*_ ((P1, P2), P3, O), the positive value revealed the introgression proportion from P3 to P2, while zero suggested no introgression. Notably, unlike the *D* statistics, the negative *f*_*d*_ value did not possess biological meaning.

We estimated the *f*_*d*_ values using the method described by Zhou et al. [64]. Briefly, the *f*_*d*_ value was calculated in each 50 kb window. The minimum site in each window was set to 100. For windows of *D* < 0, or of *D* > 0, but *f*_*d*_ > 1, the *f*_*d*_ statistic value was converted to zero. In our analysis, Yorkshire was considered as P1, Beijing Black pig as P2, and Shenxian pig as P3.

### Pairwise IBD detection

A total of 178 individuals genotyped for 31,485,393 SNPs in the whole genome served as input for the Identity By Descent (IBD) detection. IBDLD (v3.38.1) [65] was used to estimate the frequencies of shared haplotypes between Beijing Black pig and the other two different populations (Yorkshire and Shenxian pig) in different regions. The genome was divided into bins of 10,000 bp, and the number of recorded IBD between two populations was calculated per bin. Because the total number of pairwise comparisons differed between the groups, the counts of recorded IBD need to be normalized (nIBD), ranging from 0 (no IBD detected) to 1 (all pairwise individuals between two groups shared haplotype IBD). The nIBD between Beijing Black pig and one pig population was computed as follows: nIBD = cIBD/tIBD, where cIBD = count of all haplotypes IBD between Beijing Black pig and one pig group, and tIBD = total pairwise comparisons between Beijing Black pig and one pig group). Then, the relative Identity By Descent (rIBD) between Beijing Black pig and the two competing pig populations was calculated as follows: rIBD = nIBD(Yorkshire)-nIBD(Shenxian pig). We transformed the rIBD values using Z-transformation as follows: ZrIBD=(rIBD–µ)/σrIBD. The threshold for extreme IBD with Yorkshire compared with Shenxian pig was set to 2 s.d. from the mean in the far right tail of the distribution.

### Local ancestry inference in Beijing Black pig

To infer the ancestry along the Beijing Black pig genomes, we performed local ancestry implemented in LOTER (v1.0) [66]. 40 individuals of Yorkshire and 38 individuals of Shenxian pig were considered as reference populations, assuming that a haplotype of the developed breed consists of a mosaic of existing haplotypes from the two reference populations. We first assigned each allele to Yorkshire or Shenxian pig applied by LOTER, where 0 corresponded to Yorkshire while 1 corresponded to Shenxian pig. Then, we divided the genome into windows of 50 kb and calculated the frequencies of assigned two reference populations’ ancestries averaged over each non-overlapping 50 kb window. The windows with the highest or lowest 5% of the empirical distribution for averaged ancestry were considered as candidate regions with an excess of the ancestry of Shenxian pig or Yorkshire, respectively.

### Selection Signature Detection of Beijing Black pig

Since the phylogenetic tree indicated that Yorkshire and Beijing Black pig formed a monophyletic clade, Population branch statistic (PBS) [67] was performed to detect selection signatures in Beijing Black pig after divergence from Yorkshire. For each window with 50 kb size and 2 kb step, we calculated the PBS as follows:

$$ T=-\text{log}\left(1-{F}_{st}\right)$$

$$ PBS=\frac{{T}^{BY}+{T}^{BO}-{T}^{YO}}{2}$$

where *T*^*ij*^ represents the estimated branch length between i and j populations based on pairwise *F*_*st*_ estimated by VCFtools (v.0.1.16) [68]. B represents the target population (Beijing Black pig), while Y and O represent the control population (Yorkshire pig) and the outgroup (Shenxian pig), respectively. The population PBS value represents the amount of allele frequency change at a given locus since its divergence from the other two populations. For the highest 5% PBS windows, we filtered windows that coincided with previous genomic introgression regions to reduce the false positives. The remaining windows were considered to be selective sweeps.

### GWAS for meat quality traits in Beijing Black pig

Association analysis of multiple meat quality traits was performed using the single-trait linear mixed model in the GEMMA (v0.98.5) [69] based on a panel of 1,537 Beijing Black pig individuals that were genotyped on the Illumina Porcine 50 K Beadchip (See Additional file 2, Additional methods). To avoid potential false positives in multiple comparisons, the Wald statistic was employed to examine the significance of the SNP. The threshold P-value after the Bonferroni correction was 1/N, where N is the number of SNPs. In addition, the P-value of results was visualized by Manhattan plots and quantile-quantile (Q-Q) plots using R. Moreover, to avoid missing true hints of linkage, we separately extracted 10 SNPs upstream and downstream of each significant SNP to identify LD blocks using Haploview (v4.1) [70].

To further identify the candidate trait-related regions under selection in Beijing black pig, we overlapped the regions between the previous selective sweep regions and the LD blocks around tag SNPs in genome-wide association study (GWAS) analysis. The function of non-synonymous mutations in the overlapped regions were predicted by MutPred2 [71]. The protein structures with substitution amino acid were predicted by SWISS-MODEL [72].

### Electronic supplementary material

Below is the link to the electronic supplementary material.

Supplementary Material 1

Supplementary Material 2

Supplementary Material 3

## Data Availability

The dataset supporting the conclusions of this article is available in the Genome Sequence Archive (GSA) repository (PRJCA015645), https://ngdc.cncb.ac.cn/gsa/s/Vv7AncSh.

## Abbreviations

GO: Gene Ontology
GWAS: genome-wide association study
HHS: homoploid hybrid speciation
IBD: Identity By Descent
ILS: incomplete lineage sorting
IMF: intramuscular fat
KEGG: Kyoto Encyclopedia of Genes and Genomes
LD: linkage disequilibrium
NJ: neighbor-joining
PBS: population branch statistic
PCA: principle component analysis
Q-Q: quantile-quantile
rIBD: relative Identity By Descent
SNP: single nucleotide polymorphism

## References

[1] Wilkinson S, Lu ZH, Megens HJ, Archibald AL, Haley C, Jackson IJ (2013). Signatures of diversifying selection in European pig breeds. PLoS Genet.

[2] Frantz L, Meijaard E, Gongora J, Haile J, Groenen MAM, Larson G (2016). The evolution of Suidae. Annu Rev Anim Biosci.

[3] Frantz LA, Schraiber JG, Madsen O, Megens H-J, Cagan A, Bosse M (2015). Evidence of long-term gene flow and selection during domestication from analyses of eurasian wild and domestic pig genomes. Nat Genet.

[4] Bosse M, Madsen O, Megens H-J, Frantz LA, Paudel Y, Crooijmans RP (2015). Hybrid origin of European commercial pigs examined by an in-depth haplotype analysis on chromosome 1. Front Genet.

[5] Zhang W, Dasmahapatra KK, Mallet J, Moreira GR, Kronforst MR (2016). Genome-wide introgression among distantly related Heliconius butterfly species. Genome Biol.

[6] Hedrick PW (2013). Adaptive introgression in animals: examples and comparison to new mutation and standing variation as sources of adaptive variation. Mol Ecol.

[7] Racimo F, Sankararaman S, Nielsen R, Huerta-Sanchez E (2015). Evidence for archaic adaptive introgression in humans. Nat Rev Genet.

[8] Guo Y, Mao H, Ren J, Yan X, Duan Y, Yang G (2009). A linkage map of the porcine genome from a large-scale White Duroc x Erhualian resource population and evaluation of factors affecting recombination rates. Anim Genet.

[9] Li K, Ren J, Xing Y, Zhang Z, Ma J, Guo Y (2009). Quantitative trait loci for litter size and prenatal loss in a White Duroc x Chinese Erhualian resource population. Anim Genet.

[10] Li X, Yang S, Tang Z, Li K, Rothschild MF, Liu B (2014). Genome-wide scans to detect positive selection in large White and Tongcheng pigs. Anim Genet.

[11] Bosse M, Megens HJ, Frantz LA, Madsen O, Larson G, Paudel Y (2014). Genomic analysis reveals selection for Asian genes in European pigs following human-mediated introgression. Nat Commun.

[12] Ai H, Fang X, Yang B, Huang Z, Chen H, Mao L (2015). Adaptation and possible ancient interspecies introgression in pigs identified by whole-genome sequencing. Nat Genet.

[13] Zhao P, Yu Y, Feng W, Du H, Yu J, Kang H (2018). Evidence of evolutionary history and selective sweeps in the genome of Meishan pig reveals its genetic and phenotypic characterization. Gigascience.

[14] Yang W, Liu Z, Zhao Q, Du H, Yu J, Wang H (2022). Population Genetic structure and Selection Signature Analysis of Beijing Black Pig. Front Genet.

[15] Jiang Y, Yuan Z, Hu H, Ye X, Zheng Z, Wei Y (2020). Differentiating homoploid hybridization from ancestral subdivision in evaluating the origin of the D lineage in wheat. New Phytol.

[16] Tajima F (1983). Evolutionary relationship of DNA sequences in finite populations. Genetics.

[17] Cai C, Li M, Zhang Y, Meng S, Yang Y, Gao P (2020). Comparative transcriptome analyses of longissimus thoracis between pig breeds differing in muscle characteristics. Front Genet.

[18] He W, Fang X, Lu X, Liu Y, Li G, Zhao Z (2022). Function identification of bovine ACSF3 gene and its Association with lipid metabolism traits in beef cattle. Front Vet Sci.

[19] Balaban RS (1990). Regulation of oxidative phosphorylation in the mammalian cell. Am J Physiol.

[20] Ma C, Wang W, Wang Y, Sun Y, Kang L, Zhang Q (2020). TMT-labeled quantitative proteomic analyses on the longissimus dorsi to identify the proteins underlying intramuscular fat content in pigs. J Proteom.

[21] Liu JB, Cai X, Xiong H, Zhang HF (2017). Effects of feeding frequency on meat quality traits and Longissimus muscle proteome in finishing pigs. J Anim Physiol Anim Nutr (Berl).

[22] Do DN, Ostersen T, Strathe AB, Mark T, Jensen J, Kadarmideen HN (2014). Genome-wide association and systems genetic analyses of residual feed intake, daily feed consumption, backfat and weight gain in pigs. Bmc Genet.

[23] Amaral AJ, Bressan MC, Almeida J, Bettencourt C, Moreira O, Sa J (2019). Combining genome-wide association analyses and gene interaction networks to reveal new genes associated with carcass traits, meat quality and fatty acid profiles in pigs. Livest Sci.

[24] Kim JH, Ovilo C, Park EW, Fernandez A, Lee JH, Jeon JT (2008). Minimizing a QTL region for intramuscular fat content by characterizing the porcine phosphodiesterase 4B (PDE4B) gene. BMB Rep.

[25] Li X, Lee C-K, Choi B-H, Kim T-H, Kim J-J, Kim K-SJG (2010). Quantitative gene expression analysis on chromosome 6 between Korean native pigs and Yorkshire breeds for fat deposition. Genes Genomics.

[26] Wu G, Li Z, Zheng Y, Zhang Y, Liu L, Gong D (2022). Supplementing cholamine to diet lowers laying rate by promoting liver fat deposition and altering intestinal microflora in laying hens. Poult Sci.

[27] Zhuang Z, Wu J, Xu C, Ruan D, Qiu Y, Zhou S (2022). The genetic architecture of meat quality traits in a crossbred commercial pig population. Foods.

[28] Piórkowska K, Żukowski K, Ropka-Molik K, Tyra MJG (2018). Deep sequencing of a QTL-rich region spanning 128-136Mbp of pig chromosome 15. Gene.

[29] Zhang C, Wang Z, Bruce H, Kemp RA, Charagu P, Miar Y (2015). Genome-wide association studies (GWAS) identify a QTL close to PRKAG3 affecting meat pH and colour in crossbred commercial pigs. BMC Genet.

[30] Char R (2020). Pierre PJFic, biology d. The RUFYs, a family of effector proteins involved in intracellular trafficking and cytoskeleton dynamics. Front Cell Dev Biol.

[31] Zou Z, Zhang J (2016). Morphological and molecular convergences in mammalian phylogenetics. Nat Commun.

[32] Gaubert P, Wozencraft WC, Cordeiro-Estrela P, Veron G (2005). Mosaics of convergences and noise in morphological phylogenies: what’s in a viverrid-like carnivoran?. Syst Biol.

[33] Davalos LM, Cirranello AL, Geisler JH, Simmons NB (2012). Understanding phylogenetic incongruence: lessons from phyllostomid bats. Biol Rev Camb Philos Soc.

[34] Wang Z, Kang M, Li J, Zhang Z, Wang Y, Chen C (2022). Genomic evidence for homoploid hybrid speciation between ancestors of two different genera. Nat Commun.

[35] Wang Z, Jiang Y, Bi H, Lu Z, Ma Y, Yang X (2021). Hybrid speciation via inheritance of alternate alleles of parental isolating genes. Mol Plant.

[36] Abbott R, Albach D, Ansell S, Arntzen JW, Baird SJ, Bierne N (2013). Hybridization and speciation. J Evol Biol.

[37] Zhang X, Sun W, He L, Wang L, Qiu K, Yin J (2020). Global DNA methylation pattern involved in the modulation of differentiation potential of adipogenic and myogenic precursors in skeletal muscle of pigs. Stem Cell Res Ther.

[38] Wu W, Zhang J, Zhao C, Sun Y, Pang W, Yang G (2017). CTRP6 regulates porcine adipocyte proliferation and differentiation by the AdipoR1/MAPK signaling pathway. J Agric Food Chem.

[39] Roudbari Z, Coort SL, Kutmon M, Eijssen L, Melius J, Sadkowski T (2019). Identification of Biological pathways contributing to marbling in skeletal muscle to improve beef cattle breeding. Front Genet.

[40] Zhang M, Guo Y, Su R, Corazzin M, Hou R, Xie J (2022). Transcriptome analysis reveals the molecular regulatory network of muscle development and meat quality in Sunit lamb supplemented with dietary probiotic. Meat Sci.

[41] Celik O, Celik N, Gungor S, Haberal ET, Aydin S (2015). Selective regulation of oocyte meiotic events enhances Progress in Fertility Preservation methods. Biochem Insights.

[42] Handel MA, Schimenti JC (2010). Genetics of mammalian meiosis: regulation, dynamics and impact on fertility. Nat Rev Genet.

[43] Jugovic P, Hill AM, Tomazin R, Ploegh H, Johnson DC (1998). Inhibition of major histocompatibility complex class I antigen presentation in pig and primate cells by herpes simplex virus type 1 and 2 ICP47. J Virol.

[44] Dixon LK, Abrams CC, Bowick G, Goatley LC, Kay-Jackson PC, Chapman D (2004). African swine fever virus proteins involved in evading host defence systems. Vet Immunol Immunopathol.

[45] Groenen MAJGSE (2016). A decade of pig genome sequencing: a window on pig domestication and evolution. Genet Sel Evol.

[46] Cheng X, Xu C, DeGiorgio MJM (2017). Fast and robust detection of ancestral selective sweeps. Mol Ecol.

[47] Li N, Zhang X, Sun X, Zhu S, Cheng Y, Liu M (2022). Genomic insights into the evolutionary history and diversification of bulb traits in garlic. Genome Biol.

[48] Refoyo-Martínez A, da Fonseca RR, Halldórsdóttir K, Árnason E, Mailund T, Racimo FJGR (2019). Identifying loci under positive selection in complex population histories. Genome Res.

[49] Zhou D, Udpa N, Ronen R, Stobdan T, Liang J, Appenzeller O (2013). Whole-genome sequencing uncovers the genetic basis of chronic mountain sickness in Andean highlanders. Am J Hum Genet.

[50] Martin MJEj (2011). Cutadapt removes adapter sequences from high-throughput sequencing reads. EMBnet J.

[51] Li H, Durbin R (2009). Fast and accurate short read alignment with Burrows-Wheeler transform. Bioinformatics.

[52] Li H, Handsaker B, Wysoker A, Fennell T, Ruan J, Homer N (2009). The sequence Alignment/Map format and SAMtools. Bioinformatics.

[53] Faust GG, Hall IM (2014). SAMBLASTER: fast duplicate marking and structural variant read extraction. Bioinformatics.

[54] McKenna A, Hanna M, Banks E, Sivachenko A, Cibulskis K, Kernytsky A (2010). The genome analysis Toolkit: a MapReduce framework for analyzing next-generation DNA sequencing data. Genome Res.

[55] Wang K, Li M, Hakonarson H (2010). ANNOVAR: functional annotation of genetic variants from high-throughput sequencing data. Nucleic Acids Res.

[56] Tamura K, Stecher G, Kumar S (2021). MEGA11: Molecular Evolutionary Genetics Analysis Version 11. Mol Biol Evol.

[57] Kang HM, Sul JH, Service SK, Zaitlen NA, Kong SY, Freimer NB (2010). Variance component model to account for sample structure in genome-wide association studies. Nat Genet.

[58] Yang JA, Lee SH, Goddard ME, Visscher PM (2011). GCTA: A Tool for Genome-wide Complex Trait Analysis. Am J Hum Genet.

[59] Alexander DH, Novembre J (2009). Lange KJGr. Fast model-based estimation of ancestry in unrelated individuals. Genome Res.

[60] Nguyen LT, Schmidt HA, von Haeseler A, Minh BQ (2015). IQ-TREE: a fast and effective stochastic algorithm for estimating maximum-likelihood phylogenies. Mol Biol Evol.

[61] Mirarab S, Reaz R, Bayzid MS, Zimmermann T, Swenson MS, Warnow T (2014). ASTRAL: genome-scale coalescent-based species tree estimation. Bioinformatics.

[62] Sukumaran J, Holder MT (2010). DendroPy: a Python library for phylogenetic computing. Bioinformatics.

[63] Patterson N, Moorjani P, Luo Y, Mallick S, Rohland N, Zhan Y (2012). Ancient admixture in human history. Genetics.

[64] Zhou Y, Zhao X, Li Y, Xu J, Bi A, Kang L (2020). Triticum population sequencing provides insights into wheat adaptation. Nat Genet.

[65] Han L, Abney M (2011). Identity by descent estimation with dense genome-wide genotype data. Genet Epidemiol.

[66] Dias-Alves T, Mairal J, Blum MGB, Loter (2018). A Software Package to infer local ancestry for a wide range of species. Mol Biol Evol.

[67] Yi X, Liang Y, Huerta-Sanchez E, Jin X, Cuo ZX, Pool JE (2010). Sequencing of 50 human exomes reveals adaptation to high altitude. Science.

[68] Danecek P, Auton A, Abecasis G, Albers CA, Banks E, DePristo MA (2011). The variant call format and VCFtools. Bioinformatics.

[69] Zhou X, Stephens M (2012). Genome-wide efficient mixed-model analysis for association studies. Nat Genet.

[70] Barrett JC, Fry B, Maller J, Daly MJ (2005). Haploview: analysis and visualization of LD and haplotype maps. Bioinformatics.

[71] Pejaver V, Urresti J, Lugo-Martinez J, Pagel KA, Lin GN, Nam HJ (2020). Inferring the molecular and phenotypic impact of amino acid variants with MutPred2. Nat Commun.

[72] Waterhouse A, Bertoni M, Bienert S, Studer G, Tauriello G, Gumienny R (2018). SWISS-MODEL: homology modelling of protein structures and complexes. Nucleic Acids Res.

