# Supplementary material for: Genomic evidence for human-mediated introgressive hybridization and selection in the developed breed
Source: BMC Genomics. 2024 Apr 2;25:331. doi: 10.1186/s12864-024-10259-5 (PMC10986048; doi:10.1186/s12864-024-10259-5)
Supplement: Supplementary file 1 — Supplementary Material 1 [file 12864_2024_10259_MOESM1_ESM.pdf]

Additional methods

GWAS for Meat quality traits in Beijing Black pig

A total number of 1,537 longissimus dorsi muscle samples of Beijing Black pigs were sampled to perform meat quality GWAS.

For the phenotypes, there were 1,537 records for carcass weight, 1,509 records for IMF content and protein content; and 1,528 records for pH 24h. IMF content and protein content were measured using Near infra-red spectroscopy (FoodScan Meat Analyser, FOSS, Denmark), pH 24h was measured in triplicate, using a portable pH meter (Testo 205, Testo, Germany) at 24 hours after slaughtering. Simultaneously, carcass weight of each sample was recorded.

For the genotypes, all samples were genotyped using the Illumina Porcine SNP50 BeadChip. Data quality control was conducted using the PLINK (v1.90). The genotype data was filtered by the following procedures: (1) SNP call rate < 0.95; (2) minor allele frequency (MAF) < 0.01; (3) Hardy–Weinberg equilibrium p-value was $< 10^{-6}$; and (4) SNPs were located in sex chromosomes and unmapped. After quality control, there were 43,639 high-quality SNPs left.

GWAS with the single-trait linear mixed model was conducted utilizing the GEMMA (v0.98.5). The model was as follows:

$$\mathbf{y} = \mathbf{X}\boldsymbol{\alpha} + \mathbf{Y}\boldsymbol{\beta} + \mathbf{Z}\boldsymbol{\gamma} + \mathbf{e}$$

where y was a vector of the observed phenotypes; $\mathbf{X}$ was a vector of SNP genotype indicators, which was coded as 0, 1 and 2 corresponding to the three genotypes AA, AB, and BB with B being the minor allele. $\boldsymbol{\alpha}$ was the effect of marker; Variable $\mathbf{Y}$

was an incidence matrix for non-genetic fixed effects, and $\boldsymbol{\beta}$ was a non-genetic vector of fixed effects including sex and carcass weight. Variable $\mathbf{Z}$ was an incidence matrix for a vector of polygenic effects, and $\boldsymbol{\gamma}$ was a vector for residual polygenic effects with an assumed N (0, $\mathbf{G}\sigma_a^2$) distribution, where σ_a^2 was the additive genetic variance and $\mathbf{G}$ was a marker inferred kinship matrix. While $\mathbf{e}$ was a vector for random residual errors with a putative N (0, $\mathbf{I}\sigma_e^2$) distribution, where $\mathbf{I}$ was identity matrix and σ_e^2 was the residual variance.
