# Supplementary material for: Genomic evidence for human-mediated introgressive hybridization and selection in the developed breed
Source: BMC Genomics. 2024 Apr 2;25:331. doi: 10.1186/s12864-024-10259-5 (PMC10986048; doi:10.1186/s12864-024-10259-5)
Supplement: Supplementary file 2 — Supplementary Material 2 [file 12864_2024_10259_MOESM2_ESM.pdf]

Additional Figures

Figure S1 Cross validation errors for diverse k values of admixture analysis.

Figure S2 Shared variants between locally developed breed and its ancestral breeds.

(a) Venn diagram shows the overlap in the number of SNVs detected among 3 breed pigs. (b) Venn diagram shows the overlap in the number of non-synonymous mutations among 3 breed pigs. (c) The pairwise genetic differentiation coefficient (F_{st}) and polymorphism levels statistic (θπ) of 3 breed pigs. The numbers in the circles represent the θπ value of each breed, and the numbers on the dotted line represent the pairwise F_{st} values.

Figure S3 The plot for the d_{xy} statistic between two contrasting groups, including Beijing Black pig versus Shenxian pig (BS) and Beijing Black pig versus Yorkshire (BY).

Figure S4 Introgression regions from Shenxian pig to Beijing Black pig detected by f_d statistic.

Figure S5 Local ancestry inference of Beijing Black pig.

Red lines display the top 5% windows with the high ancestry of Shenxian pig and blue lines display the bottom 5% windows with the high ancestry of Yorkshire.

Figure S6 LD blocks for meat traits of Beijing Black pigs detected by GWAS.

(a): pH 24h; (b-d): protein content.

The figures will be shown in the above order as follows.

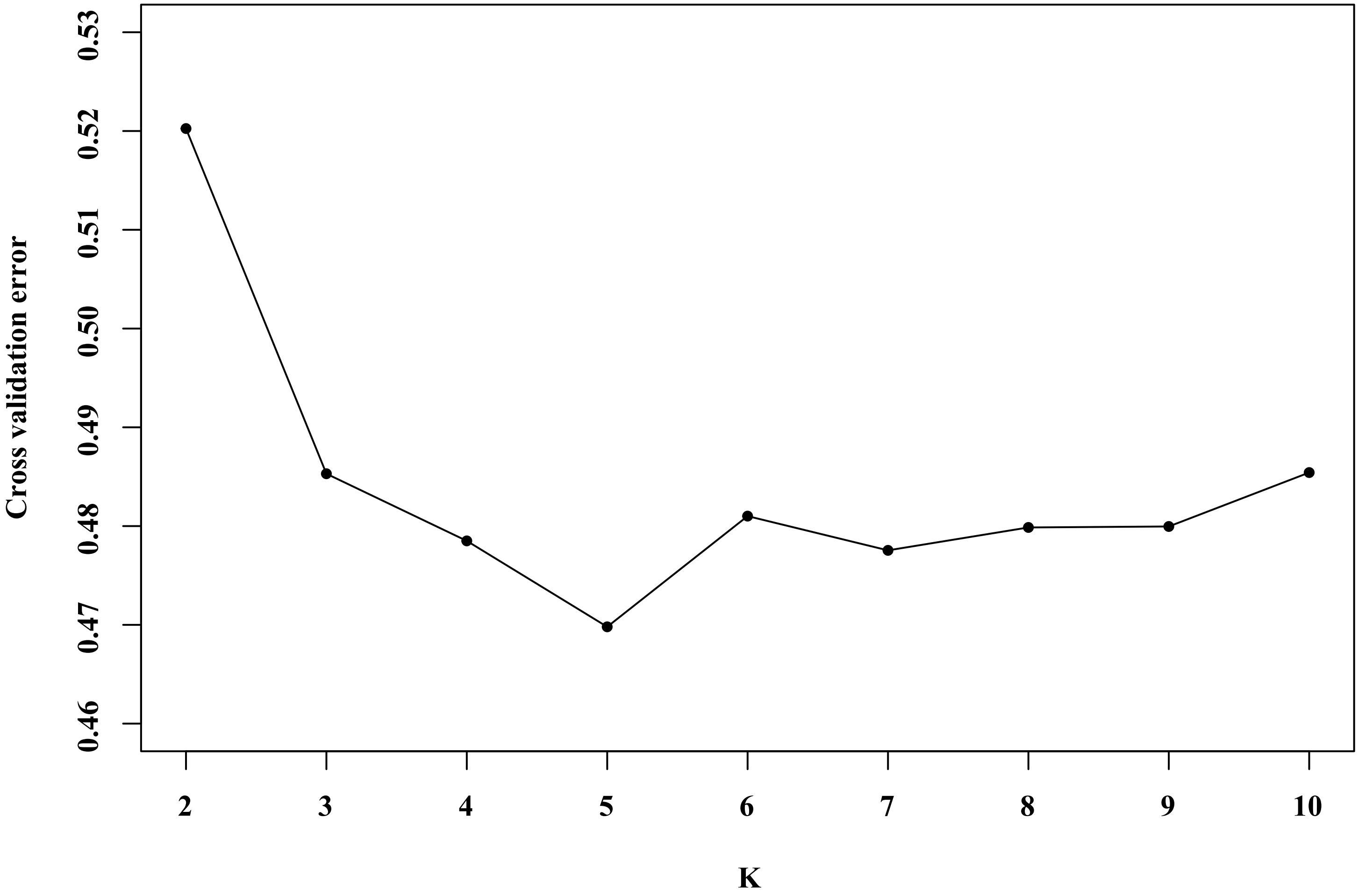

Cross validation error
0.46
0.47
0.48
0.49
0.50
0.51
0.52
0.53
2
3
4
5
6
7
8
9
10
K

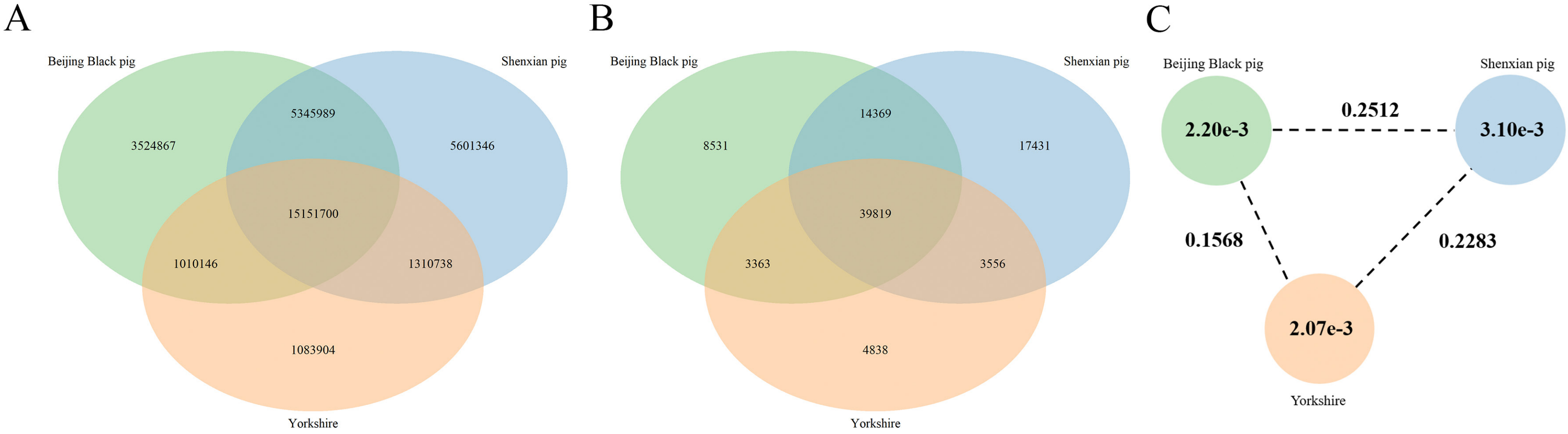
A
Beijing Black pig
Shenxian pig
5345989
3524867
5601346
15151700
1010146
1310738
1083904
Yorkshire
B
Beijing Black pig
Shenxian pig
14369
8531
17431
39819
3363
3556
4838
Yorkshire
C
Beijing Black pig
Shenxian pig
2.20e-3
0.2512
3.10e-3
0.1568
0.2283
2.07e-3
Yorkshire

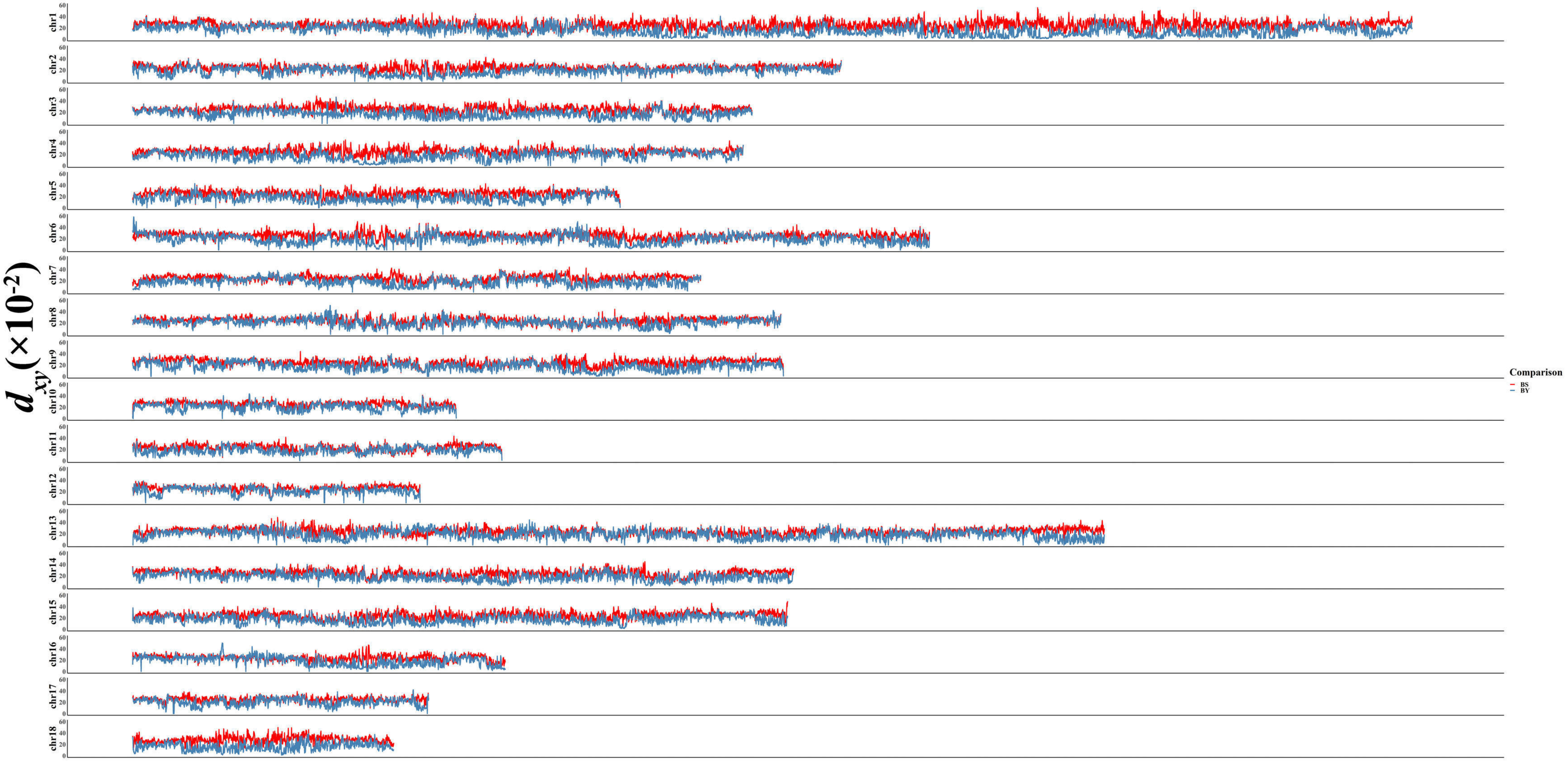
d_{xy} (×10^-2)
chr1
chr2
chr3
chr4
chr5
chr6
chr7
chr8
chr9
chr10
chr11
chr12
chr13
chr14
chr15
chr16
chr17
chr18
0
20
40
60
Comparison
BS
BY

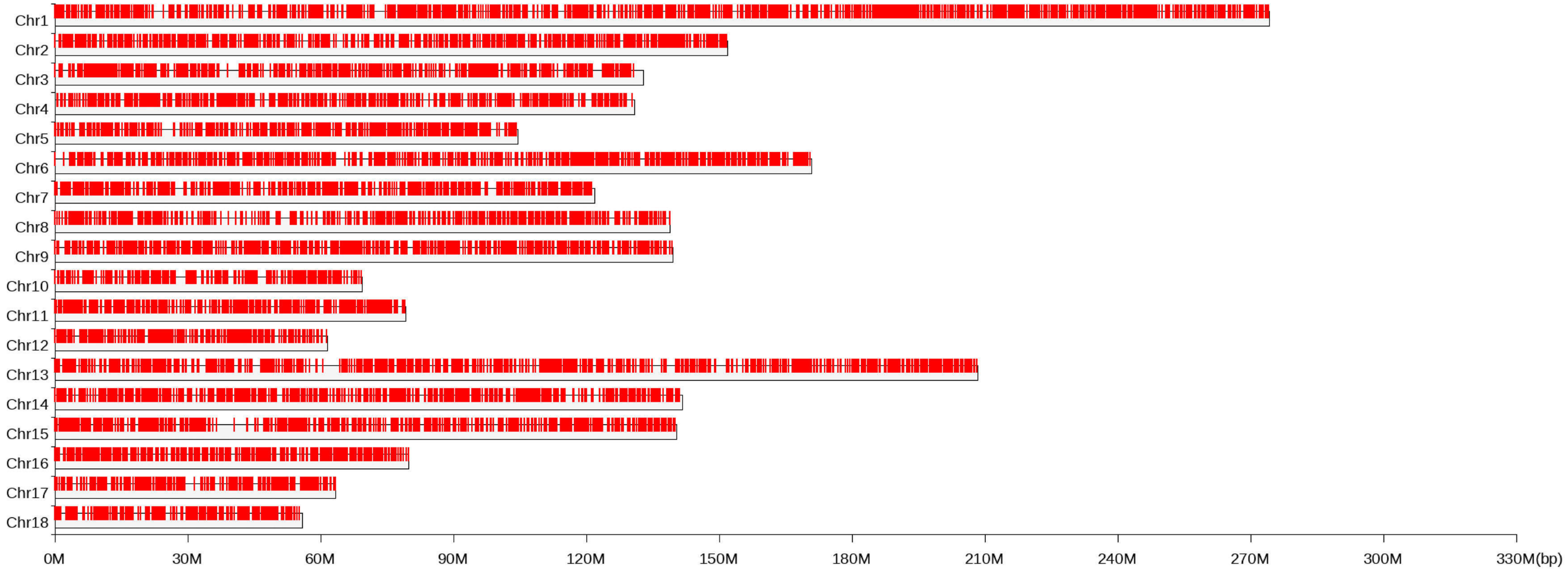
Chr1
Chr2
Chr3
Chr4
Chr5
Chr6
Chr7
Chr8
Chr9
Chr10
Chr11
Chr12
Chr13
Chr14
Chr15
Chr16
Chr17
Chr18
0M
30M
60M
90M
120M
150M
180M
210M
240M
270M
300M
330M(bp)

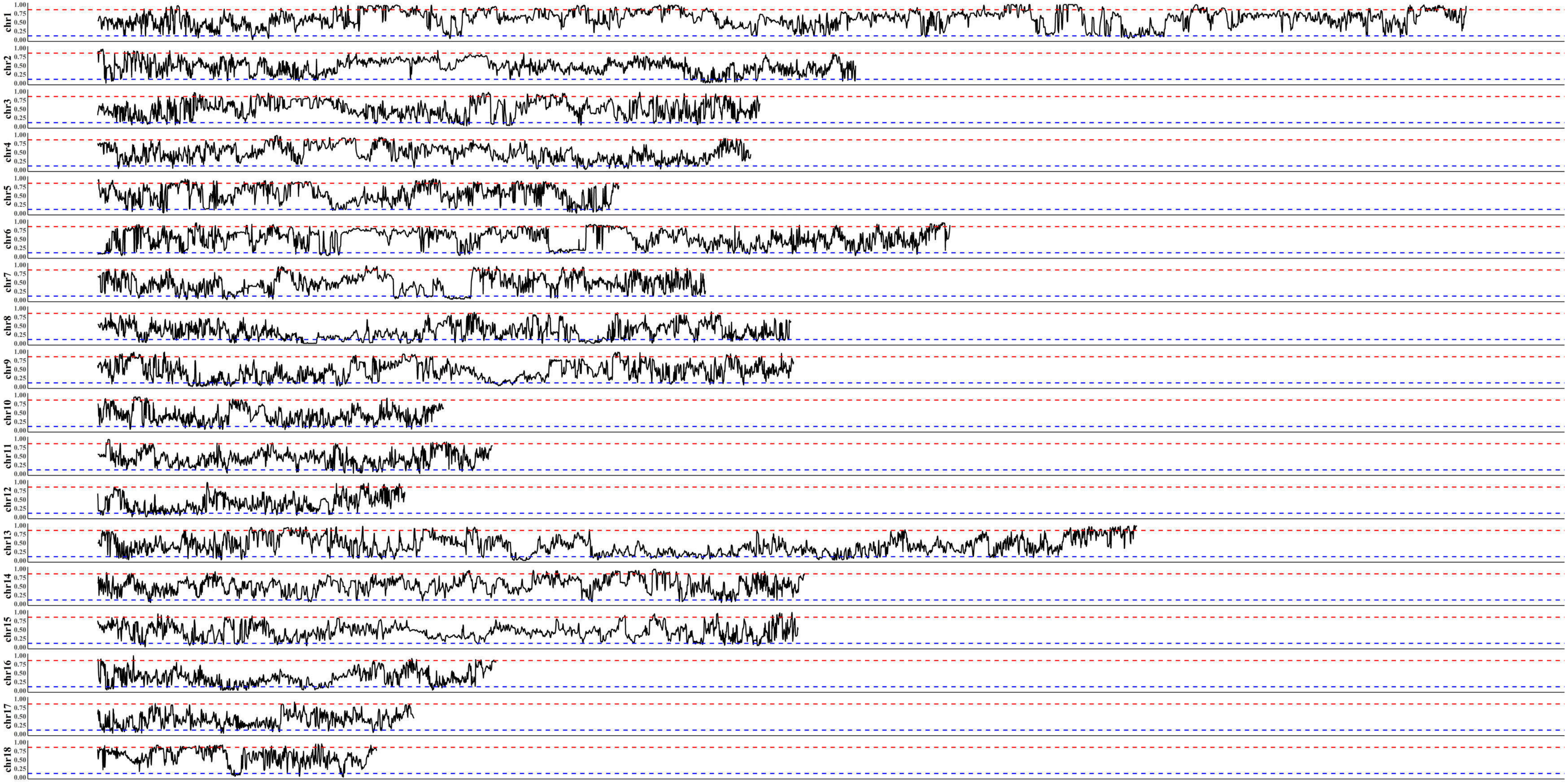
chr1
chr2
chr3
chr4
chr5
chr6
chr7
chr8
chr9
chr10
chr11
chr12
chr13
chr14
chr15
chr16
chr17
chr18
1.00
0.75
0.50
0.25
0.00

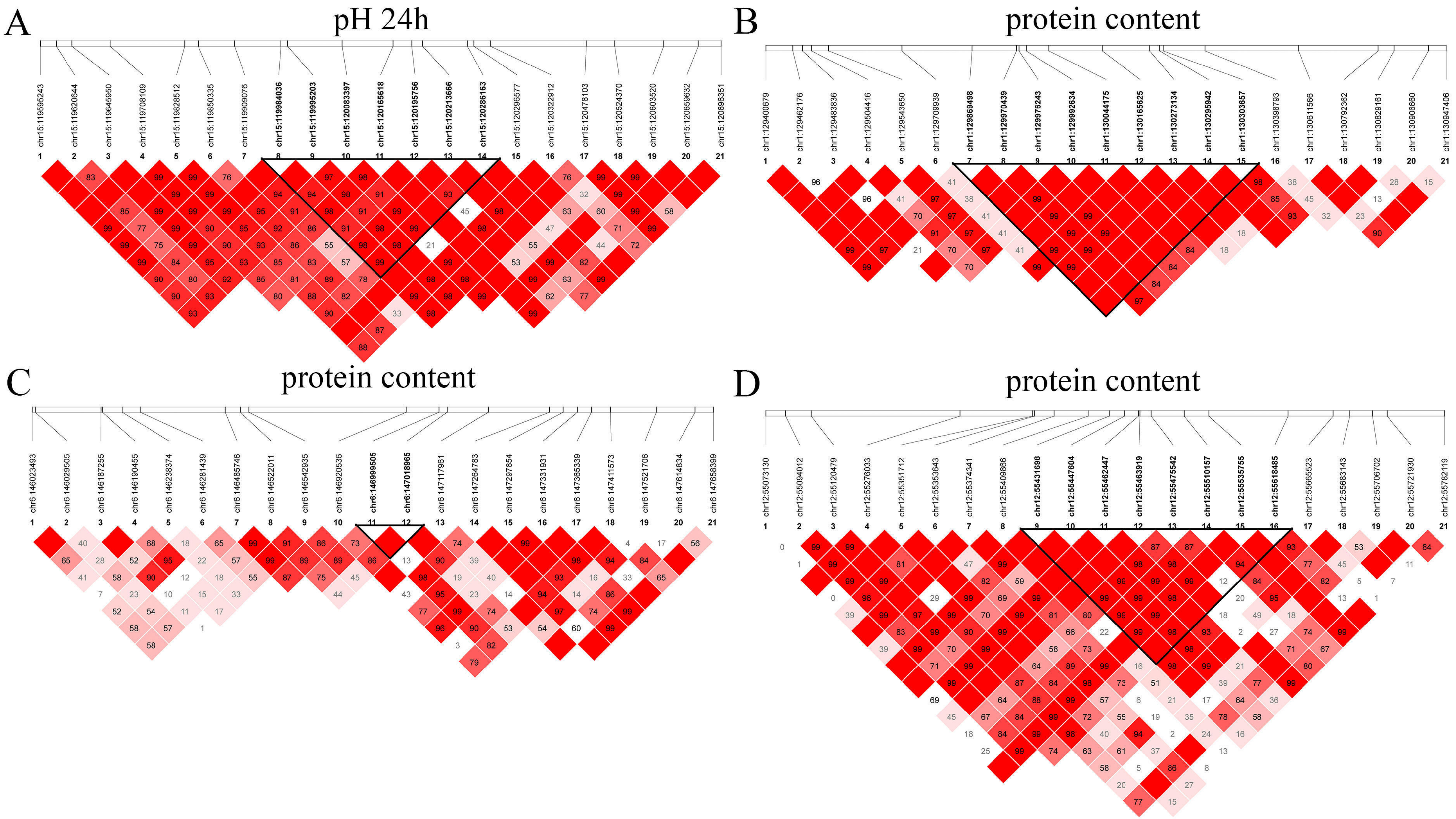
A
pH 24h
chr15:119595243
chr15:119620644
chr15:119645950
chr15:119708109
chr15:119828512
chr15:119850335
chr15:119909076
chr15:119984036
chr15:119995203
chr15:120083397
chr15:120165618
chr15:120195756
chr15:120213666
chr15:120286163
chr15:120296577
chr15:120322912
chr15:120478103
chr15:120524370
chr15:120603520
chr15:120659632
chr15:120696351
B
protein content
chr1:129400679
chr1:129462176
chr1:129483836
chr1:129504416
chr1:129543650
chr1:129709939
chr1:129869498
chr1:129970439
chr1:129976243
chr1:129992634
chr1:130044175
chr1:130165625
chr1:130273134
chr1:130295942
chr1:130303657
chr1:130398793
chr1:130611566
chr1:130792362
chr1:130829161
chr1:130906660
chr1:130947406
C
protein content
chr6:146023493
chr6:146029505
chr6:146187255
chr6:146190455
chr6:146238374
chr6:146281439
chr6:146485746
chr6:146522011
chr6:146542935
chr6:146920536
chr6:146999505
chr6:147018965
chr6:147117961
chr6:147264783
chr6:147297854
chr6:147331931
chr6:147365339
chr6:147411573
chr6:147521706
chr6:147614834
chr6:147658399
D
protein content
chr12:55073130
chr12:55094012
chr12:55120479
chr12:55276033
chr12:55351712
chr12:55353643
chr12:55374341
chr12:55409866
chr12:55431698
chr12:55447604
chr12:55462447
chr12:55463919
chr12:55475542
chr12:55510157
chr12:55535755
chr12:55618485
chr12:55665523
chr12:55683143
chr12:55706702
chr12:55721930
chr12:55782119
